# Targeting KRAS in Solid Tumors: Current Challenges and Future Opportunities of Novel KRAS Inhibitors

**DOI:** 10.3390/pharmaceutics13050653

**Published:** 2021-05-04

**Authors:** Alice Indini, Erika Rijavec, Michele Ghidini, Alessio Cortellini, Francesco Grossi

**Affiliations:** 1 Medical Oncology Unit, Fondazione IRCCS Ca’ Granda Ospedale Maggiore Policlinico, 20122 Milan, Italy; alice.indini@policlinico.mi.it (A.I.); erika.rijavec@policlinico.mi.it (E.R.); michele.ghidini@policlinico.mi.it (M.G.); 2 Department of Biotechnology and Applied Clinical Sciences, University of L’Aquila, 67100 L’Aquila, Italy; alessiocortellini@gmail.com; 3 Department of Surgery and Cancer, Imperial College London, Faculty of Medicine, Hammersmith Hospital, Du Cane Road, London W120NN, UK; 4 Medical Oncology Unit, Department of Medicine and Surgery, University of Insubria, ASST dei Sette Laghi, 21100 Varese, Italy

**Keywords:** KRAS, NSCLC, colorectal cancer, pancreatic cancer, LGSOC, endometrial cancer, AMG510, sotorasib

## Abstract

Activating mutations in RAS family proteins are found in ~25% of all human cancers. Different solid tumors are correlated with mutations in certain isoforms of RAS, with Kirsten RAS (KRAS) being the most frequently mutated isoform. Historically, KRAS has been acknowledged as “undruggable”, largely because the RAS proteins do not appear to present suitable pockets to which small inhibitory molecules can bind. However, this scenario has changed over the last years with the advent of novel KRAS inhibitors. In this review, we describe the role of KRAS mutation across different solid tumors, providing data on novel KRAS inhibitors currently under development and an updated overview of ongoing research in this field. A literature search was performed to select papers, abstracts, and oral presentation on KRAS inhibitory strategies in KRAS mutated solid tumors. Overall, the most promising therapeutic results have been obtained with molecules targeting KRAS G12C, thus paving the way for a significant therapeutic improvement in non-small cell lung cancer. Unfortunately, KRAS G12C mutation is rather uncommon in other solid tumors, namely pancreatic ductal adenocarcinoma and colorectal cancer. Several combination strategies are currently under evaluation in clinical trials, in order to bypass the resistance mechanisms responsible for the intrinsic resistance of mutated KRAS to the main therapeutic strategies adopted to date. Results suggest that the therapeutic scenario of KRAS has started to change, and further research will bring therapeutic results in this field.

## 1. Introduction

Cancer is one of the leading causes of death worldwide, accounting for an estimated 9.6 million deaths in 2018, with lung and colorectal cancers being the two most common causes of cancer-related deaths [1,2]. Despite the significant improvements in survival outcomes with the advent of targeted therapies and immunotherapy, the majority of patients with advanced/metastatic solid tumors will eventually progress on systemic treatment and die of disease [2,3]. 

The RAS proto-oncogene has been identified as a main driver of tumorigenesis in human cancers [4]. Different solid tumors are correlated with mutations in certain isoforms of RAS, with Kirsten RAS (KRAS) being the most frequently mutated isoform [5]. The frequency of KRAS mutation in the most common lethal solid tumors has led to several investigations in search of effective therapeutic approaches targeting KRAS. Historically, KRAS has been acknowledged as “undruggable”, largely because the RAS proteins do not appear to present suitable pockets to which small inhibitory molecules can bind [6]. However, over the last years, novel molecules targeting KRAS have shown promising results, suggesting that this paradigm is potentially changing with a future positive impact on therapeutic strategy [7].

In this review, we describe the role of KRAS mutation across different solid tumors. We also provide data on novel KRAS inhibitors currently under development and an updated overview of ongoing research in this field. The referenced papers were selected through a PubMed search performed on 11 March 2021 with the following searching terms: KRAS and non-small cell lung cancer (NSCLC), or colorectal cancer (CRC), or pancreatic cancer, or low-grade serous ovarian carcinoma (LGSOC), or endometrial cancer (EC). Oral presentation, abstracts, and posters presented at the American Society of Clinical Oncology (ASCO) 2020 and the European Society for Medical Oncology (ESMO) 2020 annual meetings were retrieved for preliminary data of KRAS inhibitors currently under investigation. Clinicaltrials.gov (accessed on 4 May 2021) was searched to identify ongoing clinical trials of KRAS inhibitors in solid tumors.

## 2. *KRAS* Mutation in Solid Tumors

RAS family proteins are encoded by the highly homologous genes HRAS, NRAS, KRAS4A, and KRAS4B, of which activating mutations are involved in ~25% of all human cancers [8]. Most mutations affect the KRAS isoform (~86%), which is predominant in NSCLC, CRC, pancreatic ductal adenocarcinoma (PDAC), LGSOC, and EC.

KRAS protein is a small guanosine triphosphate (GTPase) which connects cell membrane growth factor receptors to intracellular signaling pathways and transcription factors, thus playing a major role in many cellular processes [9]. RAS proteins are made of two functional domains, a G domain which binds to GTP, and a membrane-targeting domain; only after KRAS is effectively attached to the cell membrane can it be activated by binding to GTP. Once KRAS is bound to GTP, it activates more than 80 downstream signaling pathways, such as the mitogen-activated protein kinase (MAPK)–MAPK kinase (MEK), phosphoinositide 3-kinase (PI3K)–AKT–mechanistic target of rapamycin (mTOR), and rapidly accelerated fibrosarcoma (RAF)–MEK–extracellular signal-regulated kinase (ERK) [10]. KRAS also activates several transcription factors, such as ELK, JUN, and MYC, promoting crucial processes involved in cell differentiation, proliferation, transformation, and survival [10]. Mutation in KRAS impairs the intrinsic activity of GTPase, and prevents GTPase-activating proteins (GAPs) from converting GTP to guanosine diphosphate (GDP) [10]. KRAS is thus permanently bound to GTP and activates downstream signaling pathways and nuclear transcription factors, leading to sustained cell proliferation and survival.

**Figure 1:**
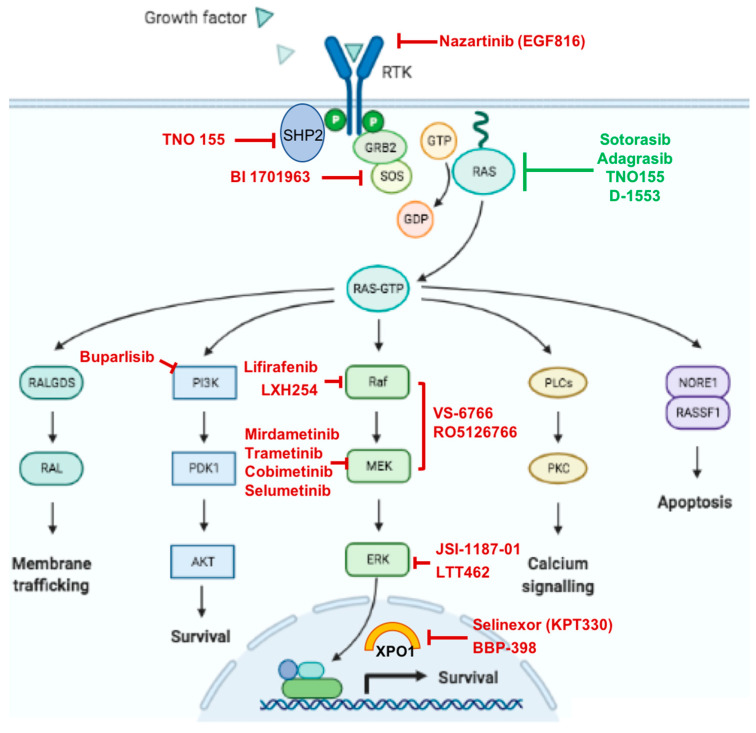
Major effector intracellular pathways activated by KRAS and an overview of the most important therapeutic compounds currently under development; the green color indicates selective KRAS G12C inhibitors.

Beyond this sustained replication property, KRAS mutation also mediates autocrine effects and crosstalk with several components in the tumor microenvironment (TME), promoting inflammation and evading the immune response [11,12]. Tumor cells expressing mutated KRAS induce the production of cytokines, chemokines, and growth factors, mediating the remodeling of surrounding stroma cells [13]. Moreover, oncogenic KRAS interacts with other mutated oncogenes and tumor suppressor genes, inducing a pro-inflammatory immunosuppressive stroma, which contributes to immune evasion and tumor progression [13]. Figure 1 summarizes the most important signaling effector pathways activated by KRAS.

The molecular characteristics of RAS proteins have made them “undruggable” due to the absence of proper binding sites, except for the GDP/GTP pocket to which RAS proteins bind very tight, making the chance to find a competitive nucleotide analogue very difficult. For this reason, much research has focused on targeting upstream or downstream proteins in the same pathways, specifically RAS modulators or mediators of RAS-specific synthetic lethality [14]. However, this approach has often turned out to be unsuccessful due to the multiple intrinsic parallel escape mechanisms of RAS pathways [13,14]. The use of KRAS as a therapeutic target in different types of solid tumors is discussed in the further sections.

## 3. Non-Small Cell Lung Cancer

### 3.1. Role of KRAS

KRAS mutations are very frequent in NSCLC, especially in smokers; they are detected in approximately 30% of adenocarcinoma in Western countries [15,16]. KRAS G12C (glycine 12 to cysteine) mutation accounts for ~50% of all KRAS mutations, and is detected in approximately 11–16% of patients with lung adenocarcinoma. Other frequently observed mutations include KRAS G12V and KRAS G12D [17]. Schleffer et al. demonstrated that co-occurring mutations in the tumor protein p53 gene (*TP53*) (39.4%), serine/threonine kinase 11 gene (*STK11*) (19.8%), kelch like ECH associated protein 1 gene (*KEAP1*) (12.9%), ATM serine/threonine kinase gene (*ATM*) (11.9%), MNNG HOS transforming gene (*MET*) amplifications (15.4%), erb-b2 receptor tyrosine kinase 2 gene (*ERBB2*) amplifications (13.8%, exclusively in G12C), *EGFR* (1.2%), and *BRAF* (1.2%) are detected in KRAS mutated NSCLC [18]. KRAS mutation seems to be an independent negative prognostic factor for survival in NSCLC [19]. Goulding et al. performed a systematic review and meta-analysis demonstrating that KRAS mutational status could be associated with poor prognosis for survival and response outcomes in advanced NSCLC patients [20]. 

Several studies have investigated the prognostic role of KRAS mutation in patients receiving immune-checkpoint inhibitors. However, to date the role of KRAS during immunotherapy is not well established. Results from a meta-analysis by Kim et al. demonstrated that immunotherapy compared to chemotherapy significantly improved OS in pretreated patients with mutated KRAS NSCLC but not in those with wild-type KRAS [21]. On the contrary, results from a retrospective study involving 530 previously treated NSCLC patients treated with nivolumab showed that KRAS status was not a reliable predictor of immunotherapy efficacy in terms of response and survival rates [22]. Chengming et al. demonstrated that mutated KRAS NSCLC showed an inflammatory phenotype with adaptive immune resistance, characterized by an increased proportion of CD8^+^ tumor-infiltrating lymphocytes (TILs) and high tumor mutational burden (TMB) [23]. An exploratory analysis of patients randomized to first line pembrolizumab vs. platinum-based chemotherapy in the KEYNOTE-042 trial, showed that patients with KRAS G12C mutation had higher programmed cell death ligand 1 (PD-L1) tumor proportion score (TPS) and TMB, compared with KRAS wild-type patients. Based on the efficacy results of immunotherapy regardless of KRAS mutational status in this trial, the authors suggested that a pembrolizumab-based treatment can be considered a valuable comparator for clinical trials of first line KRAS targeted therapy in KRAS mutated NSCLC [24]. This putative immune sensitizing feature of KRAS mutation can be partly impaired by the concomitant presence of other mutations, as loss or alteration of serine-threonine kinase 11 (*STK11*)/liver kinase B1 (*LKB1*), which acts as a genomic driver of primary resistance to immune-checkpoint inhibitors [25,26]. 

### 3.2. Therapeutic Approach

In the past, several trials have investigated the inhibition of KRAS downstream signaling pathways, including RAF/MEK/ERK and PI3K/AKT/mTOR pathways, with disappointing overall results. This approach probably failed due to the many alternative feedback mechanisms modulating these pathways [27]. Despite promising findings from a phase II study, results from the phase III SELECT-1 trial demonstrated that the MEK inhibitor selumetinib in combination with docetaxel did not improve progression-free survival (PFS) compared to chemotherapy alone in 510 KRAS mutated NSCLC patients (median PFS 3.9 months vs. 2.8 months, *p* = 0.44) [28,29]. The MEK1/MEK2 inhibitor trametinib did not improve PFS and response rates (RR) compared with docetaxel in 121 KRAS mutated NSCLC patients [30]. In the phase II study BASALT-1, Vansteenkiste et al. investigated the pan-PI3K inhibitor buparlisib in PI3K pathway-activated, relapsed NSCLC patients. The trial stopped early due to futility at the first interim analysis [31]. 

Recently, several inhibitors targeting KRAS G12C with similar covalent binding mechanisms have been investigated in clinical trials. Adagrasib (MRTX849) is a potent and selective KRAS G12C inhibitor that demonstrated a significant anti-tumor efficacy in all evaluated KRAS G12C mutated cancer models [32]. The administration of adagrasib, at a dose of 600 mg orally twice a day, has been investigated in the multi-cohort, phase I/II KRYSTAL-1 study (NCT03785249), in 110 patients with advanced solid tumors, including 79 NSCLC patients with KRAS G12C mutation, who had progressed after previous standard treatments. Preliminary efficacy and safety results of adagrasinb in the cohort of NSCLC patients were presented during the 32nd EORTC-NCI-AACR Symposium on Molecular Targets and Therapeutic in 2020: among 51 NSCLC patients evaluable for response, objective response rate (ORR) was 45% and disease control rate (DCR) was 96% [33]. Adagrasib demonstrated a manageable safety profile, the most common grade 3 or greater treatment-emergent adverse events (TEAEs) included nausea, diarrhea, vomiting, fatigue, and elevations of aminotransferase levels [33].

Sotorasib (AMG 510) is an oral KRAS G12C inhibitor that permanently blocks KRAS G12C in an inactive GDP-bound state. In preclinical analyses, this small molecule demonstrated the ability to promote tumor regression and improve the efficacy of chemotherapy and targeted agents. Furthermore, sotorasib in combination with immune checkpoint inhibitors demonstrated tumor regression in mice models [34]. CodeBreak 100 (NCT03600883) is a phase I/II study evaluating sotorasib in patients with advanced solid tumors harboring KRAS G12C mutation, who progressed after previous standard treatments. Recently, Hong et al. published the results of the phase I study with 129 patients enrolled [7]. The primary endpoint was safety, while key secondary endpoints included pharmacokinetics (PK), ORR, DCR, duration of response (DoR), and PFS. Sotorasib demonstrated notable anti-tumor activity across all 56 NSCLC patients enrolled in the trial [7]. ORR was 32% and DCR was 88%. The median time to response was 1.4 months and the median DoR was 10.9 months. The median PFS for NSCLC patients was 6.3 months. Tumor shrinkage was described in 71% of patients at the first week-6 assessment. Sotorasib showed a favorable safety profile, with no dose-limiting toxic effects or treatment-related deaths reported. Any grade treatment-related adverse events (TRAEs) occurred in 56% of enrolled patients, and only two patients (1.6%) experienced serious adverse events. The most common adverse events described were diarrhea, fatigue, nausea, vomiting, and elevations of aminotransferase levels [7].

During the 2020 World Conference on Lung Cancer (WCLC), results from the phase II CodeBreak 100 study, enrolling 126 advanced NSCLC patients, were presented. Forty-six patients experienced a confirmed response, resulting in an ORR of 37% and DCR of 80%. The median time to objective response was 1.4 months, and the median DoR was 10 months. The median PFS was 6.8 months. TRAEs of any grade were described in 70% of patients, leading to discontinuation in 9 (7.1%) patients. The most frequently reported grade 3 TRAEs were alanine aminotransferase increase (6.3%), aspartate aminotransferase increase (5.6%), and diarrhea (4.0%). There were no treatment-related deaths.

The randomized, phase III CodeBreak 200 trial (NCT04303780), comparing sotorasib WITH docetaxel in advanced NSCLC patients with KRAS G12C mutation who have progressed after platinum-based doublet chemotherapy and checkpoint inhibitor, is currently recruiting [35]. Table 1 shows the characteristics of ongoing clinical trials of KRAS mutated tumors (direct KRAS targeting), while Table 2 shows ongoing clinical trials of drugs targeting KRAS pathways. Details on the mechanisms of action of novel therapeutic compounds are reported in Table 3.

In December 2020, Amgen submitted a New Drug Application (NDA) to the U.S. Food and Drug Administration (FDA) and Marketing Authorization Application (MAA) to the European Medicines Agency (EMA) for the use in second line of sotorasib in advanced NSCLC patients harboring KRAS G12C mutation.

## 4. Pancreatic Cancer

### 4.1. Role of KRAS

KRAS is the most frequent somatic alteration among PDAC (approximately 86% of cases), with G12D and G12V variants accounting for about 80% of KRAS mutations and being the initiating event in the majority of PDAC cases [36]. Other less frequent point mutations occur on codon 13 of exon 2 (G13D, G13C, G13S, G13R) (7%), codon 61 of exon 3 (Q61H, Q61L, Q61K, Q61R) (1–2%), and codon 117 (K117) and codon 146 (A146) of exon 4 (<1%) [37]. KRAS mutation occurs early in PDAC cancerogenesis, and can also be found in preneoplastic pancreatic lesions, as pancreatic intraepithelial neoplasia (PanIN) and intraductal papillary mucinous neoplasm (IPMN) [37].

KRAS mutation in patients with PDAC tends to be associated with poor survival outcomes, regardless of disease stage [9,38]. Oncogenic KRAS regulates several biological processes in PDAC, such as metabolism modulation [39], macropinocytosis, and autophagy stimulation [40], leading to cell growth and proliferation. In addition, KRAS protein interacts with immune cells and tumor-associated fibroblasts in the TME, through paracrine secretion of chemokines, such as the granulocyte–macrophage colony-stimulating factor and IL-6, and proangiogenic factors, such as the vascular endothelial growth factor and chemokines [39,41].

Mutated KRAS proteins activate numerous signaling pathways, which play a significant role in the pathogenesis of PDAC. Activation of the ERK1-MAPK pathway contributes to chemoresistance mechanisms, as demonstrated by the high levels of ERK1/ERK2 in gemcitabine-resistant PDAC [42]. Activation of PI3K leads to the downstream activation of AKT and mTOR molecules, and the NFκB pathway, enhancing acinar-to-ductal metaplasia, pancreatic tumor cell motility, and survival [43,44]. Another pathway activated by oncogenic KRAS is RAS-like (RAL)A–RALB, which is essential for tumor initiation, endocytosis, exocytosis, and subsequent resistance to chemotherapy and radiotherapy [45]. Besides these classical pathways, many KRAS-associated proteins are involved in PDAC, including SARC, STAT3, COX2, and EGFR1 pathways, which contribute to tumor progression and metastases induced by KRAS mutated PDAC [46].

### 4.2. Therapeutic Approach

Interestingly, there has been little correlation between PDAC mutational status and the efficacy of specific targeted therapies. Attempts to use KRAS as a therapeutic target in PDAC has largely been disappointing. KRAS G12C inhibitors have only limited potential, since G12D and G12V represent the majority of KRAS mutations in PDAC, while G12C mutations are rare [36]. The molecular structure of mutated KRAS has stronger affinity for GTP and a sterically blocked active site [10]. Moreover, there is a complex relationship of different molecular drivers, redundancy of signaling pathways, activation of bypass tracks, and the simultaneous involvement of immune-modulatory mechanisms in KRAS mutated cancer cells and the TME [47,48]. 

Several strategies of KRAS inhibition have been investigated in PDAC, both through direct and indirect targeting. The most promising results came from the use of small interfering RNAs (siRNA), which mediate the suppression of KRAS mutated cells in pancreatic cells mouse models [49]. In order to be adequately delivered into tumor tissues, siRNAs need to be delivered with the aid of a vector. The Local Drug Eluter (LODER) is a biodegradable polymeric matrix that protects the siRNA and enables its local release over a period of months within the tumor tissues [50]. This approach, combined with FOLFIRINOX chemotherapy, was demonstrated to improve the overall survival (OS) of 15 patients with PDAC in a small phase I–IIa study [51]. A phase II study, the PROTACT trial, is currently evaluating the clinical efficacy of siG12D LODER in combination with chemotherapy in patients with locally-advanced PDAC (NCT1676259) [52]. Other effective approaches allowing siRNAs delivery to tumor tissue are inhibitory exosomes (iExosomes) and nanoliposomal delivery platform using 1,2-dioleoyl-sn-glycero-3-phosphatidylcholine (DOPC), which both demonstrated efficacy in several preclinical models of PDAC [53,54]. The use of mesenchymal stromal cells-derived exosomes with KRAS G12D siRNA (iExosomes) in metastatic PDAC patients with KRAS G12D mutation is currently under investigation in a phase I clinical trial (NCT03608631) [55]. Other potential strategies of direct KRAS inhibition include targeting of the RAS-binding pocket, anti-RAS vaccination, and the disruption of RAS membrane localization. To date, all these approaches have yielded disappointing results in patients with PDAC [10].

Strategies of indirect KRAS inhibition in PDAC include targeting downstream signaling pathways. MEK inhibition have provided only limited therapeutic results, due to the adaptive reactivation of MAPK pathways and multiple parallel signaling redundancy [14]. The allosteric MEK1/2 inhibitor trametinib did not improve PFS in patients with PDAC when used in combination with gemcitabine, nor did selumetinib when compared with capecitabine in gemcitabine-refractory PDAC [56,57]. Several other MEK inhibitors are currently under investigation in phase I/II trials in combination with gemcitabine, including pimasertib, refametinib, and MSC1936369B [58,59]. One of the adaptive MAPK responses to MEK inhibitors occurs through SHP2 or PTPN11, and promising data on the pharmacological inhibition of SHP2 suggest it might represent a valid therapeutic approach for the treatment of KRAS mutated tumors [59]. In addition, combined inhibition with MEK and EGFR inhibitors, or dual inhibition of the EGFR pathway, have shown positive results, however this has never translated into significant therapeutic changes [60,61]. Similarly, single targeting of PI3K, AKT, or mTOR have been unsuccessful. A translational study investigating the potential bypass mechanisms to PI3K/mTOR inhibition in KRAS mutant CRC revealed the enhancement of other signaling pathways like EGFR, ERBB2, and ERBB3 as mechanisms of resistance, suggesting that combined PI3K/mTOR and EGFR inhibitors may improve therapeutic outcomes [62]. Other targets that have been investigated include RALA and RALB, Janus kinase (JAK) 1 and 2, NF-Kb, cell cycle regulators, and molecules involved in authophagy mechanisms [10] (Table 2). 

## 5. Colorectal Cancer

### 5.1. Role of KRAS

Mutations in the *RAS* family genes are a common finding in CRC, with KRAS being the most common (85%), followed by *NRAS* (15%), and *HRAS* (1%) [63]. KRAS mutations occur in approximately 44% of metastatic CRC (mCRC), with the majority being observed in codons 12 and 13 of exon 2 (80% are G12D, G12V, G12C, G12A, and G13D), and less frequently in codon 61 of exon 3 (5% are Q61H, Q61L, and Q61R) and codon 146 of exon 4 (2% are A146T and A146V) [63]. KRAS codon 12 or 13 mutations are a major predictive biomarker for resistance to anti-EGFR therapy in patients with mCRC, thus being recognized as a negative prognostic factor [64]. Constitutive acivation of the MAPK signaling pathway is a major cause of resistance to anti-EGFR antibodies. *NRAS* mutations yield similar effects to KRAS activation and are also predictors of anti-EGFR treatment resistance [64]. Besides its predictive role for absence of response to anti-EGFR therapy, *RAS* mutation also has a negative prognostic impact in CRC, being related with right-sided colon tumors, advanced disease, poor differentiation, and presence of liver metastases [65]. 

### 5.2. Therapeutic Approach

Similarly to KRAS mutated PDAC, therapeutic strategies that have been investigated in CRC include directly targeting mutant KRAS, parallel inhibition of downstream pathways, and targeting KRAS-membrane association. Among direct KRAS-targeting strategies, sotorasib was the first specific and irreversible KRAS G12C inhibitor to show a remarkable DCR among heavily pretreated patients with KRAS mutated CRC in a phase I trial of the CodeBreak100 (NCT03600883) [7,66]. Selumetinib was tested in patients with metastatic CRC who failed previous lines of chemotherapy, but did not show an improvement in disease progression and survival, probably due to the crosstalk between PI3K and MAPK pathways [67]. Combined downstream MEK and PI3K/mTOR inhibition has shown promising activity in blocking tumor cell proliferation in CRC xenografts [68]. In this study, the combination of a MEK1/2 inhibitor (AZD6244) and a PI3K inhibitor (BEZ235) in tumor-bearing mice showed initial reduction and subsequent inhibition of tumor growth, suggesting that this combination therapy might enhance anti-tumor activity in KRAS mutant cells [68]. Targeting KRAS-membrane structure association has been investigated in the past, and showed some positive preclinical results. Treatment with EMICORON, a synthetic compound binding to G4 structures, downregulated KRAS mRNA and protein expression in CRC cell lines, and decreased tumor volume in patient-derived xenografts bearing KRAS mutation. EMICORON administration also correlated with improved efficacy of chemotherapy in CRC-bearing mice [69]. 

Other therapeutic strategies currently under investigation in CRC include targeting KRAS-membrane association, modulation of KRAS-regulated metabolic pathways, KRAS synthetic lethal interactions, and the use of immunotherapy. Inhibition of KRAS-membrane association prevents KRAS signaling and subsequent oncogenic activity: phosphodiesterase-6δ (PDEδ) inhibitors (deltarasin and deltazinone 1) showed promising activity in vitro, but their chemical properties were unstable in vivo [70]. Targeting metabolic pathways include the use of glutaminase and glyceraldehyde 3-phosphate dehydrogenase (GAPDH) inhibitors, with the aim to kill KRAS mutated cells by blocking the intracellular pathways used to sustain their metabolic need [71]. The role of immunotherapy is supported by the evidence that immune-checkpoint inhibitors are more effective in KRAS mutated NSCLC, and this combination represents an interesting therapeutic strategy as well as combination with several other targeted therapies [72]. Synthethic lethality approaches are under evaluation in phase I/II clinical trials, with MEK inhibitors combined with BCL2, AKT, and SHP2 in KRAS mutated solid tumors [73] (Table 2). 

## 6. Other Solid Tumors

### 6.1. Low-Grade Serous Ovarian Carcinoma

LGSOC is a rare type of epihelial ovarian or primary peritoneal neoplasms, representing 5–10% of all serous ovarian carcinomas [74]. LGSOC has a more indolent biologic behavior, as compared with its high-grade counterpart, however it usually shows chemotherapy resistance [74]. Activating mutations in the MAPK pathway are the most common somatic alterations (~40%), with KRAS mutation occurring in approximately 15–54% of LGSOC among different case series [75]. Inhibitors of the RAS/RAF/MEK pathway have been investigated in patients with LGSOC [76]. Selumetinib provided 80% DCR, with a median PFS of 11 months and 2-year OS of 55% in a phase II trial enrolling 52 patients with LGSOC (2 patients had BRAF mutation, and 14 patients had KRAS mutation) [77]. There have also been several case reports of patients with KRAS or *NRAS* mutated LGSOC responding to MEK inhibitors [78,79,80]. A phase Ib trial investigating the combination of trametinib (MEK1/2 inhibitor) and buparlisib (PI3K inhibitor) showed a 28.6% response rate and DCR of 76.2% in the cohort of patients with LGSOC [81]. 

### 6.2. Endometrial Cancer

EC is the most common tumor of the female genital tract in developed countries [82]. There are two types of EC: type I EC are usually low to intermediate-grade tumors, frequently showing expression of estrogen receptors, while type II EC tend to be high-grade disease, with more advanced stages at presentation [83]. KRAS mutations have been associated with type I EC (10–30% of cases), and are frequently associated with microsatellite instability (MSI) EC [84,85]. An analysis of EC patients from the Tumor Cancer Genome Atlas (TCGA) showed that KRAS mutant ECs have increased activation of estrogen signaling, by means of increased RAS/MAPK pathway signaling. This issue has potential therapeutic relevance, as it implies that anti-estrogen therapy might be needed to reach maximum efficacy for the treatment of KRAS mutant EC [86]. In a phase II trial in women with recurrent EC, selumetinib failed to demonstrate pre-trial specifications of clinical efficacy [87]. Pathway activation analysis suggests that single agent targeted therapy might not translate into significant therapeutic results. Several combination strategies have been investigated, including the combination of MEK inhibitors with Akt inhibitors, and poly-ADP ribose polymerase (PARP) inihibitors in association with Akt or PI3K/mTOR inhibitors, although with scarce clinical results.

## 7. Conclusions and Future Perspectives

KRAS mutation represents the initiating event in several types of tumors, namely NSCLC, and PDAC. On the other hand, this could be not entirely true for CRC, which are thought to be initiated by others’ mutations such as the loss of *APC*, or by mutations in β-catenin in mismatch repair deficient tumors [6]. For many years, KRAS mutations have been considered one of the most challenging targets. The poor outcome generally observed in KRAS mutated tumors and the lack of specific drugs have made KRAS the oncologists’ biggest foe. Recently, thanks to research efforts, several compounds specifically targeting KRAS have been identified. The most encouraging results have been obtained with KRAS G12C inhibition. Treatment with sotorasib and adagrasib, the most promising drugs currently under evaluation, resulted in high anti-tumor activity while maintaining a predictable and manageable safety profile, providing data supporting their approval in a short time. 

Unfortunately, KRAS G12C mutation is detected in only a small number of patients harboring KRAS mutation. Moreover, in preclinical studies, several mechanisms of acquired resistance to KRAS G12C, leading to the activation of alternative *RAS* dependent pathways, have been described. Such resistance can be mediated through on target and off target mechanisms, including *ex novo* synthesis of GTP-bound KRAS G12C, which is maintained in its active, drug-insensitive state by EGFR and aurora kinase signaling, and adaptive feedback reactivation of wild-type KRAS [88,89]. In this context, KRAS G12C loss of heterozygosity has been proposed as a potential biomarker to predict sensitivity to G12C inhibitors [90]. In order to maximize the effect of these inhibitors, several combination therapies with immune checkpoint inhibitors and inhibitors of *RAS* related pathways, including insulin-like growth factor (IGF)1 and mTOR, and receptor tyrosine kinases and PI3K, are currently under investigation [91]. Pan KRAS inhibitors, including drugs targeting son of sevenless 1 (SOS1) and SHP2, are in early phases of clinical development, either as monotherapies or in combination with MEK inhibitors (Table 1). Another promising strategy includes targeting the transcription initiation factor 4 (eIF4), which is part of an essential protein complex regulating the translation initiation of multiple oncogenic pathways, including KRAS. A phase I trial of eFT226 (zotatifin), a selective inhibitor of eIF4A, is currently recruiting patients with HER2, ERBB3, FGFR1, FGFR2, and KRAS mutant solid tumors (Table 2).

Other emerging approaches include the use of proteolysis-targeted chimeras (PROTACs) for KRAS G12C, and clustered regularly interspaced short palindromic repeats (CRISPR)/CRISPR associated protein 9 (Cas9) for KRAS G12D/V [92]. PROTACs are molecules which induce selective degradation of the target protein of interest (i.e., KRAS G12C), thus resulting in its ubiquitination and subsequent proteasomal degradation. Compared with direct inhibition, degradation could lead to a more potent effect, especially for RAS proteins, which engage complex and multiple protein–protein interactions [93]. Several efforts have been made to develop successful degrader molecules targeting KRAS G12C. However, there have been several bottlenecks in the development, mostly linked to the chemical structure of the molecules, and the choice of the binding sites and the ligase [94]. Targeted protein removal through PROTACs is highly complementary to CRISPR-based genomic strategies [95]. CRISPR/Cas9 from *S. pyogenes* has been successfully used to edit the genomes of mammalian cells using a chimeric single-guide RNA. *RAS* mutant-specific CRISPR/Cas9 induced selective in vitro growth inhibition of various cancer cell lines harboring KRAS mutations [96]. Though intriguing, to date these strategies are characterized by challenging delivery and important off-target toxicity, thus limiting their application in clinical practice [92].

The most intriguing strategy consists of the possibility to exploit the influence of KRAS on the immune system, with a potential synergistic effect of KRAS inhibition with immune checkpoint inhibitors [91]. Besides the combination of KRAS inhibitors with immunotherapy, other promising strategies include mRNA-based vaccines, and adoptive T cell therapy specifically targeting KRAS G12 D/V, which are currently under investigation in phase I/II clinical trials [92]. A phase I trial is currently underway to investigate the role of mRNA-5671/V941, encoding for KRAS *G12D*, *G12V*, *G12C,* and *G13D*, as monotherapy or combined with pembrolizumab in patients with solid tumors harboring prevalent KRAS mutations (Table 2).

Overall, the evidence supports that significant advances are upcoming in the treatment of KRAS mutant solid tumors. Further studies are required to better define resistance mechanisms, and to widen treatment opportunities with potential combination therapies.

## Figures and Tables

**Table 1:** Main characteristics of ongoing clinical trials of KRAS inhibitors in KRAS mutated tumors (source: https://clinicaltrials.gov, accessed on 11 March 2021).

Trial Name, NCT Number	Phase	Condition(s)	Drug(s)	Sample Size	Primary Endpoint(s)
CodeBreak 101, NCT04185883	Ib	KRASG12C mutated advanced solid tumors	Sotorasib (AMG510) monotherapy or in combination with miscellaneous agents *	*n* = 1003	DLTs incidence TEAEs
CodeBreak 200, NCT04303780	III randomized	KRASG12C mutated NSCLC	Sotorasib vs. Docetaxel	*n* = 650	PFS
KRYSTAL 1, NCT03785249	I/II	KRASG12C mutated advanced solid tumors	MRTX849 monotherapy or in combination with pembrolizumab, or cetuximab, or Afatinib	*n* = 391	Safety PK ORR
KRYSTAL 2, NCT04330664	I/II	KRASG12C mutated advanced solid tumors	MRTX849 + TNO155	*n* = 148	Safety PK
KRYSTAL 7, NCT04613596	II	KRASG12C mutated NSCLC	MRTX849 + Pembrolizumab	*n* = 120	ORR
NCT04585035	I/II	KRASG12C mutated advanced solid tumors	D-1553	*n* = 200	DLTs and AEs incidence, PK
NCT04627142	I	KRAS mutated CRC	BI 1701963 + Irinotecan	*n* = 95	MTD, DLTs incidence, ORR
NCT04449874	Ia/Ib	KRASG12C mutated advanced solid tumors	GDC-6036 monotherapy or in combination with miscellaneous agents **	*n* = 236	AEs incidence, DLTs incidence

* Investigational regimens include the following agents (in combination with sotorasib): MEK inhibitor, PD1/PD-L1 inhibitor, SHP2 allosteric inhibitor, Pan-ErbB tyrosine kinasse inhibitor, EGFR inhibitor, mTOR inhibitor, CDK inhibitor, and chemotherapy. ** Investigational regimens include the following agents (in combination with GDC-6036): atezolizumab, erlotinib, cetuximab, and bevacizumab. Abbreviations: AEs, adverse events; CRC, colorectal cancer; DLTs, dose limiting toxicities; NSCLC, non-small cell lung cancer; ORR, objective response rate; PK, pharmacokinetics; TEAEs, treatment emergent adverse events.

**Table 2:** Main characteristics of ongoing clinical trials of drugs targeting KRAS pathway in KRAS mutated tumors (source: https://clinicaltrials.gov, accessed on 11 March 2021).

Trial Name, NCT Number	Phase	Condition(s)	Drug(s)	Sample Size	Primary Endpoint(s)
NCT03170206	I/II	NSCLC	Palbociclib + Binimetinib	*n* = 72	MTD, safety PFS
MEKiAUTO, NCT04214418	I/II	Gastrointestinal tumors	Cobimetinib + Atezolizumab +Hydroxychloroquine	*n* = 175	MTD
NCT03065387	I	Advanced solid tumors	Neratinib + Palbociclib, or Everolimus, or Trametinib	*n* = 120	MTD
NCT04620330	II, randomized	NSCLC	VS-6766 +/− Defactinib	*n* =100	ORR
NCT03981614	III, randomized	CRC	Binimetinib + Palbociclib +/− TAS102	*n* = 112	PFS
NCT03095612	I/II	NSCLC	Selinexor (KPT-330) + Docetaxel	*n* = 59	MTD
NCT02079740	Ib/II	Advanced solid tumors	Trametinib + Navitoclax	*n* = 130	AEs incidence RR, PFS
FRAME, NCT03875820	I	NSCLC, CRC, LGSOC	Defactinib + RO5126766	*n* = 80	RP2D AEs
NCT03299088	Ib	NSCLC	Trametinib + Pembrolizumab	*n* = 42	DLTs incidence
NCT04092673	I/II	Advanced solid tumors	Zotatifin	*n* = 45	TEAEs, AEs MTD, RP2D, PK
NCT03965845	Ib/II	Advanced solid tumors	Telaglenestat (CB-839) + Palbociclib	*n* = 85	Safety, MTD, RP2D
NCT03114319	I	Advanced solid tumors	TNO155 +/− Nazartinib (EGF816)	*n* = 255	AEs incidence, DLTs incidence
NCT02407509	I	Advanced solid tumors	RO5126766 + Everolimus	*n* = 94	Safety
NCT03756818	I	Advanced solid tumors	TAK-659 + Paclitaxel	*n* = 64	AEs incidence, MTD
NCT04528836	I/Ib	KRASG12C mutated advanced solid tumors	BBP-398	*n* = 60	MTD
NCT03087071	II	Anti-EGFR refractory CRC	Panitumumab + trametinib	*n* = 84	RR
NCT04418167	I	Advanced solid tumors with MAPK pathway mutations	JSI-1187-01 +/− dabrafenib	*n* = 124	TEAEs
NCT03905148	Ib	Advanced solid tumors	Lifirafenib (BGB-283) + Mirdametinib (PD-0325901)	*n* = 75	AEs, DLTs, TEAEs ORR
NCT04263090	I/IIa	NSCLC	Rigosertib + Nivolumab	*n* = 30	MTD ORR
MAZEPPA, NCT04348045	II, randomized	Metastatic PDAC patients with disease control after I line CT	Selumetinib + Nivolumab, or FOLFOXIRI	*n* = 307	PFS
NCT02974725	Ib	NSCLC	LXH254 + trametinib, or Ribociclib, or LTT462	*n* = 331	AEs incidence, DLTs incidence
NCT03829410	Ib/II	CRC	Onvansertib (PCM-075) + FOLFIRI + bevacizumab	*n* = 44	MTD, AEs incidence, ORR
NCT04132505	I	PDAC	Binimetinib + Hydroxychloroquine	*n* = 39	MTD
STOPTRAFFIC-1, NCT04599140	Ib/II	RAS mutated MSS CRC	SX-682 +/− nivolumab	*n* = 53	MTD, DLTs
NCT03520842	II	NSCLC	Regorafenib + methotrexate	*n* = 18	PFS
NCT03948763	I	KRAS mutant NSCLC, CRC, PDAC	mRNA-5671/V941 +/− pembrolizumab	*n* = 100	DLTs, AEs, discontinuation rate
NCT04146298	I/II	RAS G12V mutant PDAC (HLA-A*11-01)	Mutant KRAS G12V-specific TCR transduced autologous T cells + antiPD1	*n* = 30	AEs incidence, ORR

Abbreviations: AEs, adverse events; CRC, colorectal cancer; CT, chemotherapy; DLTs, dose limiting toxicities; EGFR, epidermal growth factor receptor; HLA, human leukocyte antigen; LGSOC, low-grade serous ovarian carcinoma; MAPK, mitogen-activated protein kinase; MSS, microsatellite stable; MTD, maximum tolerated dose; NSCLC, non-small cell lung cancer; ORR, overall response rate; PDAC, pancreatic ductal adenocarcinoma; PFS, progression-free survival; RP2D, recommended phase II dose; RR, response rate; TCR, T cell receptor; TEAEs, treatment emergent adverse events.

**Table 3:** Overview of novel therapeutic compounds acting on KRAS signaling pathways under investigation in KRAS mutated tumors.

Compound(s) Name(s)	Molecular Target and Mechanism of Action
Sotorasib (AMG510) Adagrasib (MRTX849) D-1553 GDC-6036	KRAS G12C inhibitors
TNO155	Src homology Phosphatase 2 (SHP2) inhibitor
BI 1701963	SOS1 (panRAS inhibitor)
VS-6766 RO5126766	RAF/MEK inhibitors
Selinexor (KPT330) BBP-398	Exportin 1 (XPO1) inhibitor
Defactinib (VS-6063)	Focal adhesion kinase (FAK) and proline-rich tyrosine kinase-2 (Pyk2) inhibitor
Zotatifin (eFT226)	Eukaryotic translation initiation factor (eIF) 4A1-mediated translation inhibitor
Telaglenestat (CB-839)	Glutaminase inhibitor
Nazartinib (EGF816)	Epidermal growth factor receptor (EGFR) inhibitor
TAK-659	Dual inhibitor of spleen tyrosine kinase (SYK) and FMS-like tyrosine kinase 3 (FLT3)
JSI-1187-01 LTT462	ERK1/2 inhibitor
Lifirafenib (BGB-283) LXH254	RAF inhibitor
Mirdametinib (PD-0325901)	MEK inhibitor
Onvansertib (PCM-075)	Polo-like kinase 1 (PLK1) inhibitor
SX-682	CXC chemokine receptors 1 and 2 (CXCR1/2) inhibitors

## Data Availability

Not applicable.
